# Effects of Process Parameters on the Quality of Suantang Beef

**DOI:** 10.3390/foods11223585

**Published:** 2022-11-11

**Authors:** Fangrui Liu, Chan Wang, Cuiqin Li, Laping He, Xiao Wang, Xuefeng Zeng, Yifeng Dai

**Affiliations:** 1 Key Laboratory of Agricultural and Animal Products Store & Processing of Guizhou Province, Guizhou University, Guiyang 550025, China; 2 College of Liquor and Food Engineering, Guizhou University, Guiyang 550025, China; 3 School of Chemistry and Chemical Engineering, Guizhou University, Guiyang 550025, China

**Keywords:** Suantang beef, process parameters, texture, volatile compounds, aromaactive compounds

## Abstract

Suantang beef is a traditional delicious Chinese food cooked in Suantang (ST, a sour soup fermented by microorganisms). However, the impact of ST on beef quality is unclear, and the process of ST beef lacks unified technical standards. In the presented study, we found that the additional amount of salt, cooking time, meat thickness, and beef–ST ratio significantly affect the quality of ST beef. After optimization, it was found that when salt addition was 1%, cooking time was 3 min, meat thickness was 2 cm, and beef–ST ratio was 40%, the color determined by colorimeter, texture determined by texture analyzer, and sensory scores of beef cooked by ST were improved compared with boiled beef. ST decreased the pH value and cathepsin L activity of beef, increased the content of organic acid, and changed the protein composition of beef. ST made the beef have higher hardness, and have better chewiness and cohesion. At the same time, ST reduced the disagreeable odors of beef and improved beef flavor. In addition, 88 volatile compounds were detected in ST beef by HS-SPME/GC-MS. According to odor, threshold, and odor activity value (OAV), 24 critical aroma-active compounds were confirmed in ST beef. This study provides a basis for the potential industrialized production of ST beef.

## 1. Introduction

Guizhou Suantang (ST) is a traditional hot and sour soup in southwestern China. It has a long history and unique flavor [1]. Amongst ST products, the China Food Association has recognized Kaili Hong Suantang (HST), a conventional fermented hot and sour soup made from anaerobically fermented tomatoes and chili peppers, is a Guizhou specialty [2]. HST was named one of the top three Chinese hot pot seasonings, along with Chongqing Spicy Hot Pot Seasoning and Inner Mongolia Clear Soup Instant Lamb Hot Pot Seasoning [3]. HST is made through the microbial fermentation of nutrient-rich ingredients, such as tomatoes and peppers [2]. After fermentation, HST becomes rich in beneficial bacteria, amino acids, minerals, vitamins, and organic acids. It has crucial bioactive components, such as lycopene and capsaicin [4]. Lycopene is a polyunsaturated fat-soluble carotenoid with antioxidant properties, which can effectively reduce the incidence of prostate cancer, digestive tract cancer, and cardiovascular disease [5]. Pan [6] also found that cooking meat in HST improved its nutritional quality, volatile flavor substances, color, and textural properties, which shows that ST has good advantageous properties.

Beef is a global product and the third largest meat product in the world, accounting for about 25% of the meat market [7]. It is a kind of healthy meat with high protein and high digestibility, providing essential amino acids for human health [8]. In recent years, there have been more and more studies on the effect of processing technology on the quality of beef products. For example, adding starter to beef jerky to improve beef jerky’s nutrition, texture, flavor, and safety [9]. Different barbecue temperatures changed the brown variable of steak and the volatile aroma compounds [10]. The quality of pickled beef was improved, and the time of pickled beef was prolonged through high-pressure treatment combined with reducing sodium chloride content [11]. ST beef is a southwest characteristic food made by Guizhou HST and beef. When cooked in ST, beef has a unique taste and delicious taste. However, due to the unclear impact of ST on beef quality, the lack of unified formula and technical standards, and the sales model of only small family workshops. That hinders the development of the ST beef.

The flavor is an essential part of edible meat quality. Many studies have been done on it to understand the chemical composition of meat flavor and determine the factors that affect meat flavor quality in meat production and processing [12]. In recent years, headspace solid phase microextraction (HS-SPME) combined with gas chromatography-mass spectrometry (GC/MS) has been widely used to analyze volatile components in various foods, because of its small injection volume, convenient operation, high sensitivity, high reproducibility, being solvent-free, etc., [13]. That includes the determination of volatile compounds in beef [14,15], pork [16], and mutton [17] and their products. More than 1000 volatile compounds have been identified in beef, pork, mutton, and chicken. There are much more reports on beef than pork, mutton, or poultry [12]. However, due to the lack of research on ST beef, the effect of ST on volatile compounds in beef is unclear.

In this study, beef’s physicochemical quality and flavor were studied by optimizing the cooking process of ST beef to clarify the technological conditions of ST beef and to understand the effects of ST on beef quality and its volatile compounds. In the future, it will provide a basis for solving the problems of modern production and industrial upgrading of traditional dishes, expanding the ST beef market, and bringing good social and economic benefits.

## 2. Materials and Methods

### 2.1. Processing of Beef Samples and ST

The beef hind leg meat (topside) used is from Chinese yellow cattle aged 2–3 year (live weight 450 ± 50 kg) provided by Chongqing Hengdu Agricultural Development Co., Ltd. (Chongqing, China), transported to the laboratory in an ice box, trimmed of visible connective and adipose tissues and stored at 4 °C. The process of ST was referred to the report [1]. The details are as follows: tomato mixed with other ingredients, including glutinous rice powder (2%, *w*/*w*), salt (2%, *w*/*w*), red rice (2.5%, *w*/*w*), white wine (5%, v, w), crushed fresh red pepper (20%, *w*/*w*), ginger (8%, *w*/*w*), and garlic (4%, *w*/*w*), and the mixture was sterilized (90 °C, 15 min). Next, the bacterial cell suspensions of *Lactobacillus plantarum* NR1-7, *Bifidobacteriµm animalis* subsp. *lactis* BZ11 and *Candidautilis* were added to the ST raw mixture in a ratio of 1:1:1 for fermentation.

The manufacturing process of ST beef is as follows: the beef’s connective tissue and visible fat were manually removed. The meat was cut into 2 × 3 cm, parallel to the fiber direction. After that, soak in cooking wine 4 times the volume of beef for 10 min at 25 °C, and precook in boiling water 20 times the volume of beef for 2 min. Then pour the beef into the boiled ST to cook for a specific time, take it out, drain it, and seal the packaging with 12 × 20 cm aseptic food vacuum bag, and refrigerate at 4 °C for backup.

Boiled beef as control was treated the same way as the optimized ST beef and boiled in water, which was compared with the optimized ST beef. Both boiled beef and optimized ST beef come from the same part of the same animal.

### 2.2. Orthogonal Analysis

According to pre-experiments, the ST-to-water mass ratio was 1:8 for preparing ST-beef. The base cooking parameters were as follows: salt addition 1.5%, cooking time 5 min, meat grain thickness 3 cm, and beef–ST ratio 30%. Based on the base cooking parameters, the effects of salt amount, cooking time, thickness of meat grains, and beef–ST ratio on ST beef were investigated by one factor at a time experiment (OFAT). When one of the process parameters was studied, the other basic process parameters remained unchanged. For example, when investigating the impact of salt amount, the cooking time, thickness of meat grains, and beer ST ratio on ST beer remain unchanged, which are 5 min, 3 cm, and 30%, respectively.

According to the OFAT tests, L_9_(3^4^) orthogonal tests were conducted to determine the effects of each factor on ST beef using the yield and sensory evaluation as indexes to arrive at the optimal combination. A table of the factors and levels for the orthogonal test design is shown in Table 1. Then, the optimal combination was verified.

### 2.3. Yield

The yield of the ST beef sample in the optimization process was measured according to the method described by Gök, et al. [18] with modifications. The yield of the beef sample can be obtained via Equation (1):

(1) Yield (%)=M1/M0×100% 

where M0 is the weight of the beef sample before cooking (g), and M1 is the weight of the beef sample after cooking (g).

### 2.4. Color Measurement in CIE System

The color of beef samples (ST beef in the process of optimization, optimized ST beef, and boiled beef) was determined by colorimeter HP-2132 (Beijing fourth Generation Zhongyi Co., Ltd., Beijing, China). The CIE standard light source D65 was used along the fiber direction, the observation angle was 10°, and the diameter of the measuring area was measured by 8 mm. The instrument is calibrated with a white standard board. Colors are described as L* (luminance), a* (red), and b* (yellow). The sample is in triplicate, and the surface of each sample is measured three times. 

### 2.5. Water Content, pH, and Texture Determination

The water content was measured following Wang, et al. [19]. The details are as follows: 5 g of beef samples were taken and the moisture content was determined by a rapid moisture tester (MB-35, OHAUS, Parsippany, NJ, USA). Each sample was measured three times.

Beef pH determination was referred to the report [7]. The mixture of 5 g sample and 45 mL distilled water were homogenized (XHF-DY, Ningbo Xinzhi Biotechnology Co., Ltd., Ningbo, China) at 5000 r/min for 30 s, and the final pH of beef sample was determined immediately with pH agent (PHS J-3F, Chengdu Century Ark Technology Co., Ltd., Chengdu, China).

The beef samples in the OFAT, the optimized beef samples, and the beef samples in the control group were cut perpendicular to the fiber direction and then cooled to room temperature after processing according to the sample processing method. The hardness, elasticity, chewiness, cohesion, and adhesion of beef were measured using a texture analyzer (CT3, AMETEK, Brookfield, WI, USA). The test mode was TPA, the target value was 4 mm, the interval was 5 s, the load of the trigger point was 10 g, the test speed was 1 mm/s, the prediction speed was 1 mm/s, the inversion speed was 1mm, the data frequency was 100 p/s, and the load unit is 10,000 g. Each sample was randomly measured by three points, and the average was obtained.

### 2.6. Sensory Evaluation

Sensory evaluation was performed according to the report [20] with small modifications. Sixteen panelists were selected based on their experience in sensory evaluation. Team members (8 males and 8 females, aged 22–35) assessed the appearance, taste, smell, texture, of ST beef in specific rooms with constant temperature, no noise and odor, and sufficient brightness. All reviewers had a brief discussion and training at the beginning of the sensory evaluation. Each team member evaluates three sample duplicates in random order and asks for a value between 1 and 9, where 9 is highly acceptable, and 1 is extremely unacceptable. After each test, the evaluator gargled with an appropriate amount of warm water. The sensory score was the sum score of appearance, taste, smell, and texture divided by four.

### 2.7. Extraction and Activity Determination of Cathepsin L

The determination of cathepsin L activity was according to the reference [21]. Fat and connective tissue were removed from the beef at 4 °C. The chopped meat was mixed with 4 volumetric extraction buffers (20 mM sodium acetate buffer and 5 mM L-Cys, pH 6.0) and homogenized under 7000 r/min for 15 s (XHF-DY, Ningbo Xinzhi Biotechnology Co., Ltd.). The activity of cathepsin L in the crude enzyme solution was determined with Z-Phe-Arg-MCA as substrate. One unit of enzyme activity was defined as 1 nmol AMC released per minute at 30 °C.

### 2.8. Analysis of the Protein Composition

Water-soluble, salt-soluble, and insoluble proteins of the ST beef samples were extracted, refer to references [22]. The extracted protein was observed by sodium dodecyl sulfate-polyacrylamide gel electrophoresis (SDS-PAGE). The method has been changed according to the reference [23]; 5% concentrated gel and 12% separation gel were used to separate proteins. Gels were run at 25 mA until the tracking dye reached the bottom of the well, removed, and stained. The gels were stained overnight with 0.1% coomassie brilliant blue-R250 dissolved in 40% ethanol and 10% acetic acid. Destaining was performed using 25% ethanol and 10% acetic acid.

### 2.9. Volatile Profile Analysis via HS-SPME Combined with GC-MS

Volatile profile analysis via HS-SPME combined with GC-MS was referred to our previous report [24]. Pegasus HRT 4D Plus instrument (LECO Corporation, St. Joseph, MI, USA) was used to analyze the volatile compounds in ST, optimized ST beef, and boiled beef. Put a total of 5 g equal sample into a 20 mL glass headspace vial and seal it with a spacer. Then 50 × 30 μm divinylbenzene/carboxyl/poly (dimethylsiloxane) (DVB/CAR/PDMS) coated fibers (Supelco, Inc., Bellefonte, PA, USA) were introduced into the top space of the vial at 60 °C for 30 min. The fibers were then injected into the injection port of Pegasus GC-HRT 4D Plus and desorbed at 250 °C for 3 min. Volatile compounds were separated by using DB-WAX capillary column (30 m × 0.25 mm × 0.25 μm). Volatile compounds were separated by SQ 456. The heating procedure is as follows: initial 40 °C for 3min, heating up to 150 °C at the rate of 5 °C/min, holding 1 min, heating again at the rate of 3 °C/min to 200 °C, having 1 min, and finally heating to 230 °C and keeping 6 min. Helium was used as a carrier gas. GC operates in shunt mode (5:1) with a column flow rate of 6 mL/min. The operating conditions of MS include EI as ionization method, electron energy 70 eV, interface temperature 250 °C, transmission line temperature 280 °C, ion source temperature 200 °C, and quadrupole temperature 150 °C. Volatile compounds detected in ST, optimized ST beef, and boiled beef using GC-MS were compared with previously published data, the National Institute of Standards and Technology’s MS library (search version 1.6), and Wiley library (New York, 320k compound, version 6.0). 2-Methyl-3-heptanone was used as an internal standard for semiquantitative analysis. The relative percentages of the detected peaks were obtained by peak area normalization. The estimated concentration of each volatile in the sample was calculated according to [25]. The odor activity value (OAV) described the contribution of each volatile compound. OAVs were calculated as a ratio of the concentration to the odor detection threshold. Values of odor detection thresholds were derived from the literature data [26]. Compounds with OAVs greater than or equal to 1 were considered essential contributors to odor.

### 2.10. Statistical Analysis

The experiments were conducted in triplicate (n = 3), and all data were reported as means ± standard deviation (SD). Statistical analyses were performed using SPSS 25.0 (IBM SPSS Statist cs, Chicago, IL, USA). ANOVA with Tukey’s multiple range T-test was applied to assess significance (*p* < 0.05). OriginPro 2021 (Origin, Inc., Princeton, NJ, USA) was used for graph plotting.

## 3. Results and Discussion

### 3.1. Effect of Salt Addition on the Quality of ST Beef

**Figure 1:**
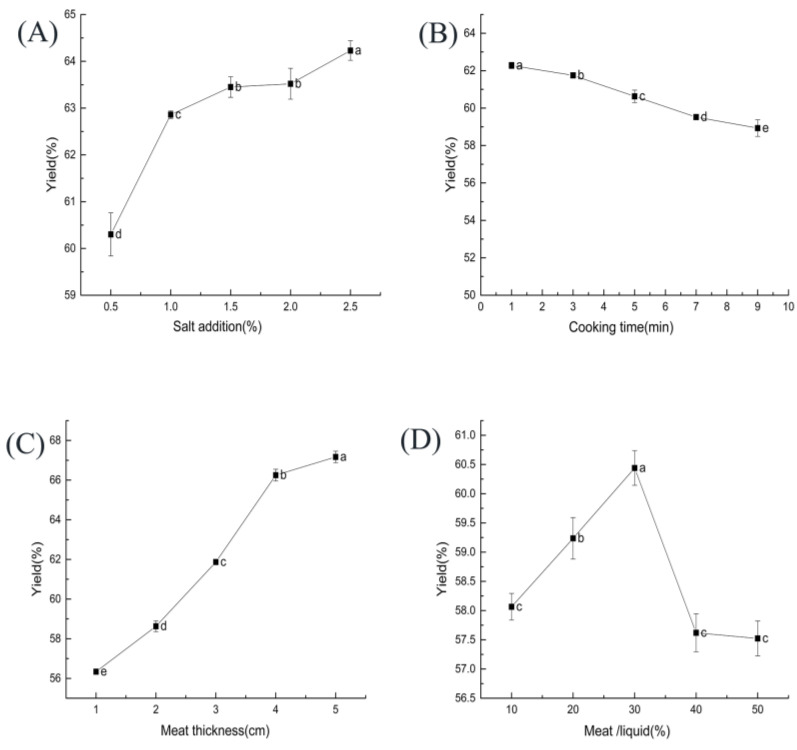
Effect of each factor on the yield of ST beef: (**A**) salt addition (based on ST mass); (**B**) cooking time; (**C**) meat thickness; (**D**) beef–ST ratio. Different letters above each bar mean significant differences.

As shown in Figure 1A, with the increase in salt addition from 0.5% to 2.5%, ST beef production increased rapidly at first and then slowly. That may be because high salt conditions cause muscles to contract through osmotic pressure, increasing the ionic strength of muscle protein gels [27]. The gel strength increase improved the protein’s water retention, thus increasing the yield.

**Figure 2:**
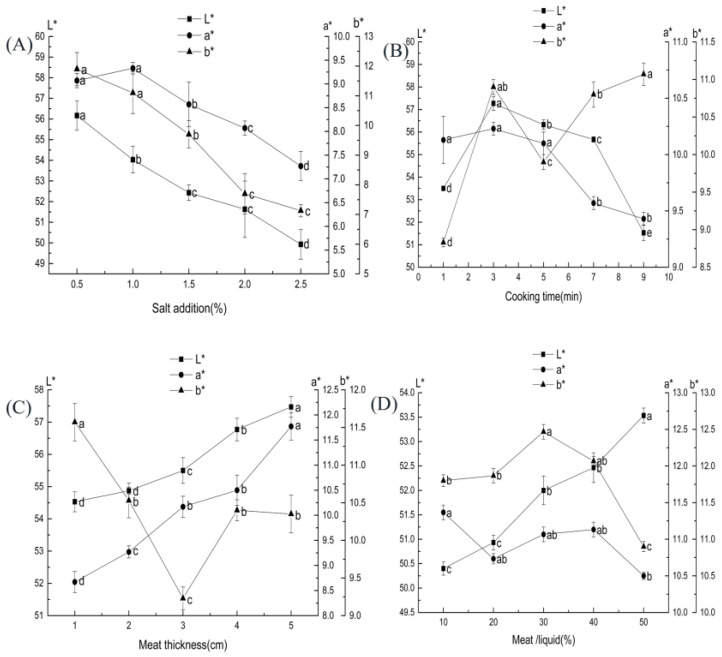
Effect of each factor on the color of ST beef: (**A**) salt addition (based on ST mass); (**B**) cooking time; (**C**) meat thickness; (**D**) beef–ST ratio. Different letters above each bar mean significant differences.

The L* and b* values gradually decreased with increasing salt addition are shown in Figure 2A. The decrease in L* values was similar to that of Horita, et al. [28] when they studied desalted Frankfurt sausages. This outcome is probably because the increased concentration of salt solution increased the muscle tissue’s ionic strength and water-holding power of the muscle tissue, which decreases the muscle tissue’s visual brightness [29]. When the salt level was over 1%, the a* value decreased significantly (*p* < 0.05). A study found that when the amount of salt in pickled beef decreased from 0.5% to 2.0%, the a* value in beef first increased and then decreased [30], which is similar to our result trend.

**Figure 3:**
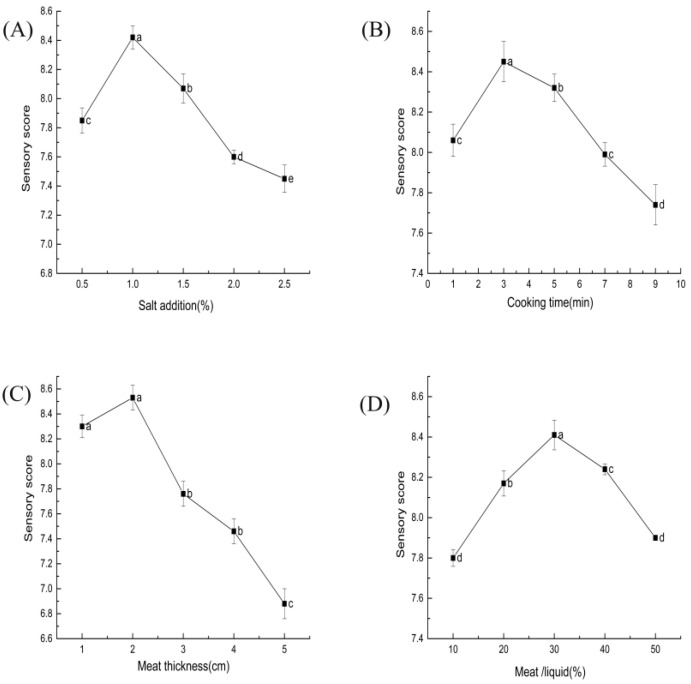
Effect of each factor on the sensory of ST beef: (**A**) salt addition (based on ST mass); (**B**) cooking time; (**C**) meat thickness; (**D**) beef–ST ratio. Different letters above each bar mean significant differences.

The sensory quality of ST beef was significantly affected by salt content (*p* < 0.05) in Figure 3A. The right amount of salt effectively improved the product’s color by promoting the penetration of sugars and nitrites into the muscle’s interior; it also brought a good flavor and taste experience [31]. The product tasted too salty when excessive salt was added and had poor flavor and salt-soluble protein breakdown [32].

As shown in Table 2, beef’s hardness, chewiness, and stickiness increase with salt added. The elasticity increases at first and then decreases, and the viscous change is irregular within the measuring range. The textural properties of the conditioned reconstituted beef products, such as hardness, elasticity, chewiness, and cohesiveness, increase significantly (*p* < 0.05) with increasing salt addition [33]. Salt affects the solubility of myogenic fibrin (salt-soluble protein), improving water retention [34], thus improving the product’s hardness, chewiness, and other structural properties.

### 3.2. Effect of Cooking Time on the Quality of ST Beef

The effect of cooking time on the yield of ST beef is shown in Figure 1B. The yield gradually decreased as the cooking time increased (*p* < 0.05). Some studies have found that meat loses fluid during cooking, resulting in textural changes and loss in cook yield [35].

The effects of cooking time on the L*, b*, and a* values of ST beef are shown in Figure 2B. As the cooking time increased, the L* value initially increased and then decreased, the a* value showed a decreasing trend, and the b* value changed in a more complicated way; it rose, then reduced, and then increased (*p* < 0.05). The color change of the meat products during cooking is related to the myoglobin content, solubility, and the release of metal ions [36]. The L* and b* values of cooked pork were significantly higher than those of fresh pork (*p* < 0.01), whereas the a* value decreased significantly (*p* < 0.01) [37]. The change in the value of b* is related to the oxidation of meat fat. In contrast, the decrease in a* value is associated with the oxidation of bright red oxygenated myoglobin to reddish-brown high-iron myoglobin [38].

As shown in Figure 3B, the sensory scores tended to increase and decrease as the cooking time increased. A cooking time of around 3 min had relatively high sensory scores. The cooking process causes water loss, muscle fiber shrinkage, and protein denaturation, resulting in less hardness and chewiness [35].

As can be seen in Table 2, beef hardness, cohesiveness and chewiness tended to increase with increasing cooking time. Beef cohesiveness and elasticity were the greatest at 1 min of cooking time and then decreased and stabilized after that (*p* > 0.05). The change in elasticity was related to the degree of muscle protein denaturation. A study of vacuum-cooked soy beef found that shear force, hardness, chewiness, and elasticity increased at first and then decreased with the increase in cooking time [39].

### 3.3. Effect of Meat Thickness on the Quality of ST Beef

As shown in Figure 1C, the thickness of the meat blocks had a significant effect (*p* < 0.05) on the yield of ST beef. As the thickness of the meat blocks increased, the yield gradually increased (*p* < 0.05). In another study, the yield of reconstituted pork blocks prepared from small pieces of meat (2–3 cm) was significantly higher than that of products prepared from large pieces of meat (4–5 cm) [40], which is different from our study. The difference in outcome may be related to the raw materials studied.

The effect of meat block thickness on ST beef is shown in Figure 2C. As the thickness of the meat block increased, the L* and a* values gradually increased, and the b* value showed an initial decrease and then increased. The main factor for the change in meat color was the denaturation of hemoglobin [41]. The increase in L* value may be due to the increase in the thickness of the meat pieces. The rise in fat and light reflection leads to an increase in luminance value.

The sensory evaluation of the thickness of the meat grains on ST beef is shown in Figure 3C. Higher sensory scores were obtained when the thickness was 2 cm. The difference between the meat thicknesses of 1 and 2 cm was insignificant (*p* > 0.05). In another study, the appearance and overall palatability of reconstituted pork cubes made of smaller pieces of meat (2–3 cm) were substantially better than those of other meat cubes [40].

The effect of meat thickness on the texture of ST beef is shown in Table 2. Beef hardness, gelatinousness, elasticity, and chewiness decreased significantly (*p* < 0.05) with increasing meat thickness. Meat thickness significantly reduced the hardness, elasticity, and chewiness of meat (*p* < 0.05) and significantly increased the cohesiveness of meat (*p* < 0.05) [42].

### 3.4. Effect of Beef–ST Ratio on the Quality of ST Beef

The effect of the beef–ST ratio on the yield of ST beef is shown in Figure 1D. The yield reached the highest value at 30% beef–ST ratio. As the beef–ST ratio increases, the surface area of the beef in contact with the broth increases, which leads to a rise in the absorption ratio. When the beef–ST ratio increases to a certain level, too much of the cooking liquid is absorbed into the meat, which causes some damage to the muscle fiber structure, reduces water retention, and decreases the yield [43].

The effects of beef–ST ratio on the L*, b*, and a* values of ST beef are shown in Figure 2D. As the beef–ST ratio increased, the L* value gradually increased, the b* value initially increased and then decreased (*p* < 0.05), and the a* value gradually reduced. The increase in L* values was due to the increased free water content as more cooking liquor entered the meat, which caused changes in light scattering on the beef surface. Tang, et al. [43] showed that the L* value of seasoned duck breast increased and then decreased as the meat-liquid ratio increased.

The effect of beef–ST ratio on the sensory score of ST beef is shown in Figure 3D. The sensory score initially increased and then decreased with an increasing beef–ST ratio (*p* < 0.05). The 30% beef–ST ratio had a relatively high sensory score. This result indicates that an appropriate ratio of material to liquid can help improve sensory quality.

The effect of beef–ST ratio on the textural properties of ST beef is shown in Table 2. A significant decrease (*p* < 0.05) in hardness and gelatinousness was observed as the beef–ST ratio increased. This outcome may be due to the increase in moisture, which affects the texture of the beef. The variation tendency in beef cohesiveness, elasticity, and chewiness was unclear.

### 3.5. Effect of Beef–ST Ratio on the Quality of ST Beef

As shown in Table 3, the main effects of the factors on sensory score were in the order: C > A > B > D (meat thickness > salt addition > cooking time > beef–ST ratio). The optimal combination was A2B2C2D3. The main effects of the factors on yield were in the order: A > B > D > C (salt addition > cooking time > beef–ST ratio > grain thickness). The optimal combination was A2B3C2D3. According to the ANOVA of the orthogonal results in Table 4, the amount of salt addition, cooking time, and meat thickness significantly affected the sensory score of ST beef soup (*p* < 0.05). 

In contrast, salt addition, cooking time, meat thickness, and beef–ST ratio significantly affected the yield (*p* < 0.05). The verification test of the sensory score was carried out according to the results of the ANOVA. The final result was that A2B2C2D3 scored 8.5 better than the other groups, and A2B2C2D3 was the optimal combination. The sensory score of ST beef under optimal conditions was 8.58, and the yield was 70.12%. These values were higher than the scores in experiments 2 and 4. Therefore, the optimal condition for beef processing in ST was 1% salt addition, 3 min cooking time, 2 cm meat thickness, and 40% beef–ST ratio.

### 3.6. Quality Analysis of ST Beef and Boiled Beef

#### 3.6.1. Physical and Chemical Qualities of ST Beef and Boiled Beef

As shown in Table 5, the physical and chemical quality scores of ST beef were mostly higher than those of boiled beef. The pH of ST beef was lower than that of boiled beef. It decreased the pH value of beef, increased the content of organic acid in meat, and enriched beef’s taste. Moreover, the ST group had higher hardness, gelatinous properties, chewiness and cohesiveness, and lower elasticity than boiled beef. These changes may be related to the decrease in pH, which causes the protein molecules in the meat to cross-link and form a dense mesh structure, resulting in higher hardness, chewiness, and cohesiveness [44]. High cohesiveness means better texture. That may also be related to the activity of cathepsin L in beef. Cathepsin L is a major cathepsin in the maturation process, which can hydrolyze various components of a myofibril of pacific cod [45]. It was found that cathepsin L activity in ST beef was significantly lower than in boiled beef. Cathepsin L plays an essential role in myofibrillar protein degradation in vivo and in vitro [21], so ST makes beef have higher hardness, chewiness, and cohesion. Cooking in ST affected L* values and had little effect on a* and b* values. This outcome is in line with the results of Pan [6]. ST reduced the brightness of the beef, which was related to the presence of pigmented substances in ST.

#### 3.6.2. Protein Composition and SDS-PAGE of ST Beef and Boiled Beef

The effects of ST processing on the contents of water-soluble protein, salt-soluble protein, and insoluble protein in beef are shown in Table 5. There was no significant difference in the total protein content between ST beef and boiled beef. However, it is worth noting that beef’s water-soluble protein and salt-soluble protein decreased after ST cooking. This phenomenon may be due to the decrease of pH and cathepsin L activity in beef after ST processing, which made the protein in ST beef have better cohesion so that the protein cannot be extracted completely when using buffer solution [21]. This result corresponds to the measured texture result.

**Figure 4:**
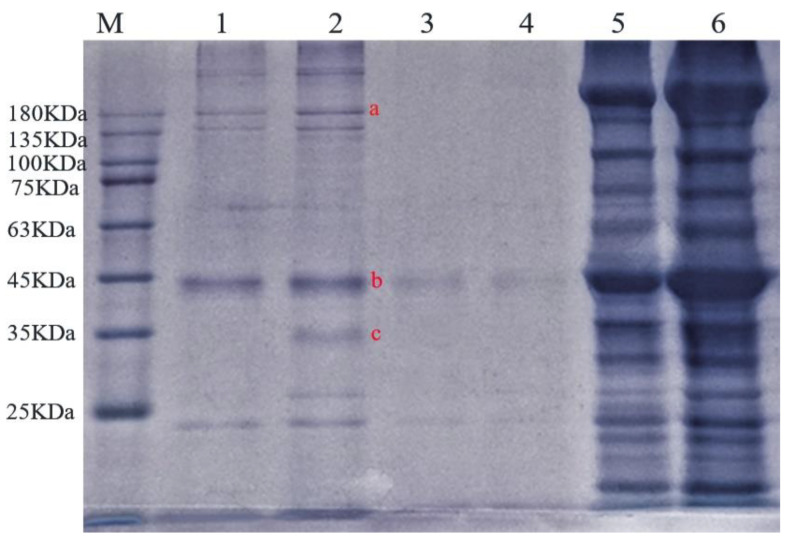
Effect of ST on the protein composition of beef: 1, 2 swimming lanes are ST beef and boiled beef water-soluble protein; 3, 4 swimming lanes are ST beef and boiled beef salt-soluble protein; 5, 6 swimming lanes are ST beef and boiled beef insoluble protein. Different letters denote different proteins.

SDS-PAGE electrophoresis of water-soluble, salt-soluble, and insoluble proteins in ST beef and boiled beef is shown in Figure 4. The water-soluble protein of beef changed obviously after being cooked by ST. The band range of 135–180 kDa molecular weight in lane 1 becomes shallow. That was maybe an incomplete extraction process, causing a band concentration to decrease; b and c bands did not appear after ST cooking, which may be that the ST prompts endogenous proteases to hydrolyze this protein. After extraction, the concentration of salt-soluble protein was low. The band is shallow, while the concentration of insoluble protein is high and the band deep, which may be due to the degradation of meat protein, protease, and peptidase, and some form large aggregates with a decrease in pH value [46]. The results of SDS-PAGE electrophoresis were consistent with those of protein content determination.

#### 3.6.3. Volatile Compounds in ST Beef and Boiled Beef

A total of 169 volatile compounds were quantified, including 50 hydrocarbons, 29 esters, 30 alcohols, 8 sulfides, 4 phenols, 11 ketones, 18 aldehydes, 5 acids, and 14 other compounds (Table S1). Hydrocarbons, esters, and alcohols are the main volatile compounds in our samples. To further examine the contribution of these 169 volatile carbides to the overall aroma characteristics of ST beef, their OAV were calculated, taking into account their concentration and odor thresholds (Table S1).

**Figure 5:**
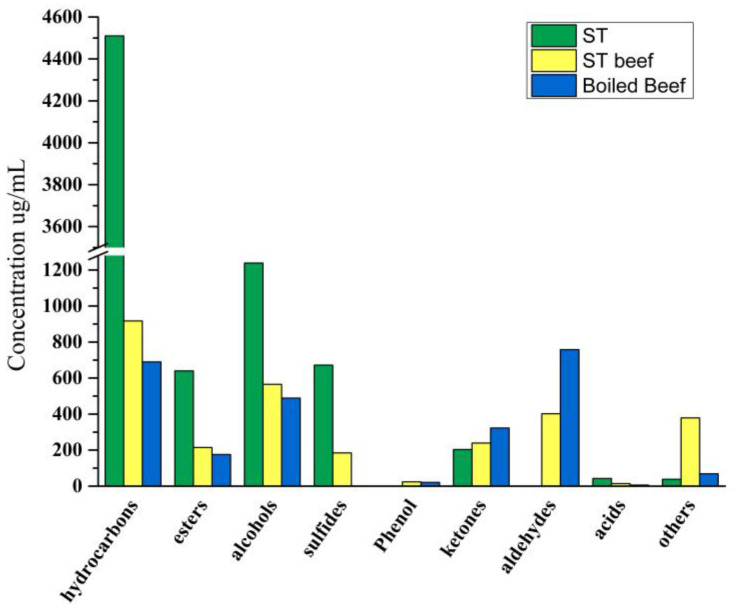
Changes in volatile compounds content in ST, ST beef, boiled beef.

Figure 5 illustrated the changes in volatile compounds in ST, ST Beef, and boiled beef. The hydrocarbon concentration in beef increased from 689.940 μg/100 g to 917.386 μg/100 g after ST cooking, combined with Table S1, hydrocarbons such as copaene, β-sesquiphellandrene, β-bisabolene, β-elemene, zingiberene increased significantly. Wang, et al. [1] found that HST has high levels of terpenes, including β-sesquiphellandrene, β-myrcene, β-elemene, β-bisabolene, α-pinene, α-farnesene, D-limonene, copaene, and α-curcumene, were mainly derived from raw materials and excipients, such as tomatoes, red peppers. At the same time, the concentration of esters in beef increased from 689.940 µg/100 g to 917.386 μg/100 g after cooking in ST.

Esters are derived from the esterification of carboxylic acids and alcohols. Esters with short-chain acids have a fruity and sweet taste, but long-chain acids produce a fatty odor [47]. Notably, beef produces two characteristic esters after cooking in ST, linalyl acetate and 4-terpinenyl ester of isobutanoic acid. Among them, linalyl acetate has a higher OAV value, which can be considered the main contributor to the aroma of ST beef. At the same time, the content of some esters decreased significantly, such as isoamyl acetylacetate, α-terpinyl acetate, and linalyl propionate. It was maybe that ST inhibited the volatilization of these esters in beef.

Alcohols are mainly produced by the oxidative decomposition of lipids [48] because of their high odor threshold and are considered to contribute little to the aroma of meat samples [49]. The carbon chain length can produce a unique aroma [50]. This study showed that the alcohol content in beef increased from 489.134 μg/100 g to 565.497 μg/100 g. The contents of geraniol, 2-heptanol, linalool, (-)-terpene-4-ol, and 1-hexanol in ST beef increased compared with the control group. Among them, geraniol and linalool are high, with sweet and pleasant flower fragrance [51], and linalool found in ham, salchichón, shoulder, and cecina contributes to flowery and citrus aroma [52]. Their content and OAV value increased significantly, which could be considered the primary source of ST beef and ST.

Aldehydes could impart a pleasant fruity and fatty sensorial note to meat products [53]. It is the main component of beef volatile flavor [54]. The aldehyde concentration in beef reduced from 758.444 μg/100 g to 402.461 μg/100 g after cooking in ST, while the aldehyde content in the ST was as low as 0.808 µg/100 g. Aldehydes are derived from the oxidation, degradation, or strecker degradation reactions of amino acids in meat fat [55], which explains the low aldehyde content in the ST. Aldehydes are readily reduced to alcohols or oxidized to acids in the presence of microorganisms [56], which may account for the lower aldehyde content and the increased alcohol content. Nonanal, octanal, benzaldehyde, and lemongrass aldehyde were detected in both beef and ST beef showing high concentrations and OAV. These substances provided sweetness to the beef and rose, citrus, and oil aromas that added to the smell of the meat [57]. Benzaldehyde was found to provide floral and almond flavors in dry-cured foal sausage [58], not from lipid oxidation but the metabolism of aromatic amino acids [59]. The results show that ST both adds flavor and retains the characteristic aroma substances of beef.

It is worth noting that the sulfide concentration increased from 0 μg/100 g to 184.782 μg/100 g after cooking in ST, no sulfide was detected in the beef, and the sulfide concentration increased after cooking in the ST. The high concentrations of diallyl disulfide and OAV in the ST beef correspond to the high concentrations of diallyl trisulfide and diallyl disulfide in the ST, which are due to the addition of garlic to the ST for fermentation. And one of the precursors of garlic flavor is s-(2-propenyl)-l-cysteine sulfoxide [60]. This compound is synthesized several times in the presence of the enzyme alliinase to form sulfide flavoring substances. Domínguez, et al. [57] found that the detection of sulfide in sausage samples may be related to the use of garlic as the ingredient of the meat product.

The concentration of ketones in beef decreased from 323.152 μg/100 g to 238.984 μg/100 g after being boiled in ST. It is worth noting that although the concentration of ketones in beef decreased after ST cooking, the types of ketones in ST beef increased, in which 2-nonanone, 2-undecanoneand acetoin showed OAV > 1. Petričević, et al. [61] found that 2-nonanone contributes to a dry-cured ham aroma with floral/fruity/blue cheese aroma. In addition, beef after ST cooking presented significantly decreased concentration of 2,3-octanedione, which was related to the formation of a rotten odor of 2,3-octanedione [62].

There are not many phenols and acid compounds in various products. It is worth noting that phenols are not detected in ST. These substances only exist in beef and ST beef. The concentration of eugenol increased from 19.108 μg/100 g to 21.323 μg/100 g after ST cooking, and the OAV was very high. Cloves generally bring eugenol with a similar and spicy aroma. It provides aroma and taste and increases beef’s mellow thickness [63]. Eugenol is considered one of the main contributors to the aroma of ST beef. Low-chain organic acids have an essential effect on the characteristic aroma of meat products because of their low odor threshold [64]. ST produced three kinds of acids by microbial fermentation; the concentration was 42.654 μg/100 g. The acid concentration of beef after ST cooking increased from 5.965 μg/100 g to 14.122 μg/100 g; among them, the acetic acid concentration increased significantly, corresponding to the decrease in pH value of ST beef. Some studies have found that acetic acid has a vinegar flavor and contributes to a mature aroma [65], which plays a vital role in developing the typical aroma of fermented sausage [64,66].

For other kinds of compounds, high concentrations of 4-allylanisole and cis-anethole were detected in both beef groups. The concentration of 4-allylanisole annua increased significantly from 40.087 μg/100 g to 349.339 μg/100 g after ST cooking. 4-Allylanisole annua is the main volatile component of the fennel plant, which can provide aroma [50]. The OAV value of 4-allylanisole annua is also very high, which can be considered one of the main contributors to the aroma of ST beef. It is worth noting that 2-pentylfuran decreased after the meat was cooked with ST. Rancidity is associated with valeraldehyde and 2-pentyl furan (metal, green, soil, legumes) [67].

## 4. Conclusions

ST improved the color determined by colorimeter, texture determined by texture analyzer, and sensory scores of beef. The best processing conditions were obtained by orthogonal design: salt addition 1%, cooking time 3 min, meat thickness 2 cm, beef–ST ratio 40%. Under this condition, cooking beef with ST had highest sensory score. ST decreased the pH value and cathepsin L activity of beef, increased the content of organic acid, changed protein composition in beef, and increased the hardness, chewiness, and cohesion in beef. In addition, beef boiled by ST produces more key aroma components with high OAV, enhancing the beef’s flavor. Moreover, beef cooking through ST also can reduce the count of unfriendly odor components in beef.

## Figures and Tables

**Table 1:** Factor level of orthogonal experiment.

Level	Factors			
	A Salt Addition/%	B Cooking Time/min	C Meat Thickness/cm	D Beef–ST/%
1	0.5	1	1	20
2	1	3	2	30
3	1.5	5	3	40

**Table 2:** Effect of each factor on texture.

Factor	Amount Added	Hardness (g)	Cohesiveness	Springiness (mm)	Adhesiveness (g)	Chewiness (mJ)
Salt addition (%)	0.5	1974 ± 3.54 ^b^	0.65 ± 0.05 ^a^	2.31 ± 0.06 ^b^	1352 ± 2.97 ^c^	28.5 ± 0.24 ^c^
	1	2017 ± 3.56 ^b^	0.58 ± 0.03 ^b^	2.36 ± 0.03 ^a^	1268 ± 2.92 ^c^	31.3 ± 0.21 ^b^
	1.5	2036 ± 3.21 ^b^	0.63 ± 0.03 ^a^	2.36 ± 0.03 ^a^	1372 ± 3.06 ^bc^	31.4 ± 0.20 ^b^
	2	2066 ± 3.28 ^b^	0.64 ± 0.05 ^a^	2.38 ± 0.05 ^a^	1521 ± 3.00 ^ab^	31.6 ± 0.29 ^b^
	2.5	2317 ± 3.60 ^a^	0.56 ± 0.06 ^b^	2.18 ± 0.05 ^c^	1606 ± 3.18 ^a^	35.6 ± 0.24 ^a^
Cooking time (min)	1	1855 ± 3.19 ^c^	0.65 ± 0.05 ^a^	2.44 ± 0.06 ^a^	1223 ± 2.52 ^c^	29.4 ± 0.30 ^b^
	3	2056 ± 3.44 ^b^	0.62 ± 0.03 ^ab^	2.15 ± 0.13 ^b^	1375 ± 2.66 ^b^	29.3 ± 0.31 ^b^
	5	2138 ± 3.63 ^b^	0.59 ± 0.05 ^b^	2.18 ± 0.05 ^b^	1305 ± 2.61 ^bc^	29.7 ± 0.17 ^b^
	7	2349 ± 3.50 ^a^	0.60 ± 0.06 ^b^	2.17 ± 0.03 ^b^	1418 ± 2.66 ^b^	30.2 ± 0.20 ^b^
	9	2367 ± 3.36 ^a^	0.61 ± 0.07 ^b^	2.13 ± 0.03 ^b^	1553 ± 2.86 ^a^	32.3 ± 0.18 ^a^
Meat thickness (cm)	1	1990 ± 3.38 ^a^	0.64 ± 0.05 ^bc^	2.58 ± 0.03 ^a^	1351 ± 2.57 ^a^	31.2 ± 0.35 ^a^
	2	1868 ± 3.31 ^a^	0.60 ± 0.06 ^c^	2.59 ± 0.06 ^a^	1216 ± 2.37 ^b^	27.7 ± 0.20 ^b^
	3	1549 ± 3.24 ^b^	0.65 ± 0.06 ^b^	2.49 ± 0.06 ^b^	1059 ± 2.80 ^c^	25.9 ± 0.25 ^c^
	4	1359 ± 2.92 ^c^	0.76 ± 0.05 ^a^	2.36 ± 0.11 ^c^	1107 ± 2.36 ^c^	27.7 ± 0.23 ^b^
	5	1060 ± 2.57 ^d^	0.75 ± 0.05 ^a^	2.32 ± 0.07 ^c^	829 ± 2.58 ^d^	20.7 ± 0.31 ^d^
Beef–ST (%)	10	2316 ± 3.17 ^a^	0.60 ± 0.05 ^b^	2.37 ± 0.06 ^b^	1484 ± 2.45 ^a^	34.3 ± 0.23 ^b^
	20	2155 ± 3.45 ^b^	0.68 ± 0.06 ^a^	2.49 ± 0.06 ^a^	1569 ± 2.46 ^a^	37.9 ± 0.22 ^a^
	30	2052 ± 3.05 ^b^	0.62 ± 0.06 ^b^	2.15 ± 0.03 ^d^	1372 ± 2.37 ^b^	30.2 ± 0.35 ^c^
	40	1850 ± 2.93 ^c^	0.61 ± 0.06 ^b^	2.32 ± 0.06 ^b^	1222 ± 2.62 ^c^	28.7 ± 0.29 ^d^
	50	1681 ± 2.64 ^d^	0.54 ± 0.06 ^c^	2.25 ± 0.07 ^c^	1050 ± 2.31 ^d^	23.7 ± 0.22 ^e^

Note: Data are expressed as means ± SD; different letters in the same column for the same factor indicate significant differences (*p* < 0.05).

**Table 3:** Orthogonal experiment results of formula and technology of ST beef.

Test Number	A	B	C	D	Sensory Evaluation	Turnout Rate
1	1	1	1	1	8.21	53.61
2	1	2	2	2	8.54	58.74
3	1	3	3	3	7.71	68.49
4	2	1	2	3	8.43	69.7
5	2	2	3	1	7.64	58.61
6	2	3	1	2	8.41	64.83
7	3	1	3	2	7.44	54.64
8	3	2	1	3	8.54	53.69
9	3	3	2	1	8.23	58.98
Sensory evaluation						
k1	8.15	8.03	8.39	8.03	Optimal solution A2B2C2D3	
k2	8.16	8.24	8.40	8.13		
k3	8.07	8.12	7.60	8.23		
R	0.09	0.21	0.80	0.20		
Turnout rate						
k1	60.28	59.32	57.38	57.07	Optimal solution A2B3C2D3	
k2	64.38	57.01	62.47	59.40		
k3	55.77	64.10	60.58	63.96		
R	8.61	7.09	5.10	6.89		

Note: A: salt addition, B: cooking time, C: meat thickness, D: beef–ST ratio; K1, k2, and k3 are the average index values of each factor at levels 1, 2, and 3, respectively; R is the difference between the maximum and minimum average index values of each factor at each level.

**Table 4:** Analysis of variance of orthogonal test results.

Testing Indicators	Factors	Sum of Square	Df	Mean Square	F-Value	Significance
Sensory evaluation	Salt addition	0.848	2	0.424	10.822	**
	Cooking time	1.503	2	0.752	19.174	**
	Meat thickness	0.662	2	0.331	8.449	**
	Beef–ST ratio	0.082	2	0.041	1.043	
	Error	0.706	18	0.039		
Turnout rate	Salt addition	478.969	2	239.485	507.957	**
	Cooking time	263.091	2	131.545	279.013	**
	Meat thickness	117.247	2	58.624	124.343	**
	Beef–ST ratio	50.449	2	25.224	53.502	**
	Error	8.486	18	0.471		

Note: significance: **, *p* < 0.05, significant.

**Table 5:** Physicochemical properties of cooked beef.

Indicators	ST Beef	Boiled Beef
pH	6.71 ± 0.06 ^a^	7.28 ± 0.08 ^b^
Sensory evaluation	8.41 ± 0.09 ^a^	7.35 ± 0.14 ^b^
Moisture content/%	66.35 ± 0.25 ^a^	65.73 ± 0.17 ^a^
Cathepsin L activity/U	102.43 ± 3.64 ^a^	132.73 ± 5.55 ^b^
Water-soluble proteins mg/mL	1.25 ± 0.01 ^a^	2.81 ± 0.15 ^b^
Salt-soluble proteins mg/mL	0.05 ± 0.01 ^a^	0.15 ± 0.01 ^b^
Insoluble protein mg/mL	3.08 ± 0.25 ^a^	9.39 ± 1.16 ^b^
Total protein mg/mL	17.19 ± 0.15 ^a^	16.62 ± 0.77 ^b^
Hardness (g)	2020 ± 3.38 ^a^	1618 ± 3.54 ^b^
Springiness (mm)	3.12 ± 0.12 ^a^	3.25 ± 0.17 ^a^
Adhesiveness (g)	1408 ± 3.02 ^a^	1122 ± 2.16 ^b^
Chewiness (mJ)	49.17 ± 1.08 ^a^	35.80 ± 0.56 ^b^
Cohesiveness	0.72 ± 0.10 ^a^	0.66 ± 0.07 ^a^
L*	49.70 ± 0.22 ^a^	51.77 ± 0.38 ^b^
a*	9.70 ± 0.21 ^a^	9.57 ± 0.24 ^a^
b*	12.60 ± 0.75 ^a^	12.50 ± 0.53 ^a^

Note: ST beef is an optimized sample. Data are expressed as means ± SD; different letters in the same line indicate significant differences (*p* < 0.05).

## Data Availability

Data is contained in the main article and Supplementary Materials.

## Supplementary Materials

The following supporting information can be downloaded at: https://www.mdpi.com/article/10.3390/foods11223585/s1, Table S1: Identification results and relative content of volatile compounds.
