# Supplementary material for: Effects of Process Parameters on the Quality of Suantang Beef
Source: Foods. 2022 Nov 11;11(22):3585. doi: 10.3390/foods11223585 (PMC9689645; doi:10.3390/foods11223585)
Supplement: Supplementary file 1 [file foods-11-03585-s001.zip › foods-1947193-supplementary.pdf]

Supplementary Material

1 Supplementary Tables

Table S1 Identification results and relative content of volatile compounds

Compounds	CAS	Concentration (μg/100g)			Odor Threshold (mg/kg)	OAV	
		ST	ST beef	Boiled beef		ST beef	Boiled beef
α-Bergamotene	17699-05-7	-	16.317±6.321	-	100[1)]	＜1	-
β-Sesquiphellandrene	20307-83-9	689.791±13.56	80.096±15	-	40[1)]	＜1	-
Trans-α-Bergamotene	13474-59-4	-	1.235±0.123	3.262±0.264	NF	-	-
Copaene	3856-25-5	42.996±2.665	15.695±4.321	-	NF	-	-
Sabinene	3387-41-5	-	14.711±5.321	8.504±0.580	NF	-	-
Zingiberene	495-60-3	729.22±26.229	254.428±23.43	-	NF	-	-
(±)-Camphor	464-48-2	3.247±0.095	-	-	NF	-	-

D-Limonene	5989-27-5	110.383±9.13	-	358.499±14.849	0.22[1)]	-	16.295
α-Humulene	6753-98-6	-	-	4.349±0.321	0.16[1)]	-	＜1
Hexadecane	544-76-3	-	0.846±0.093	1.463±0.210	300[1)]	＜1	＜1
Pentadecane	629-62-9	-	57.135±11.098	87.986±11.722	NF	-	-
Terpinolene	586-62-9	17.71±0.768	15.143±6.466	-	NF	-	-
Camphene	79-92-5	229.072±10.529	5.873±0.967	-	NF	-	-
Cyclene	508-32-7	3.132±0.094	-	-	NF	-	-
(4E,6Z)-2,6-dimethylocta-2,4,6-triene	7216-56-0	-	3.85±0.564	-	NF	-	-
Germacrene D	23986-74-5	79.536±5.378	8.752±0.957	1.366±0.093	1.8[1)]	＜1	＜1
Alloaromadendrene	25246-27-9	21.952±1.2613	-	-	NF	-	-
δ-Cadinene	483-76-1	59.925±4.932	10.569±6.466	-	NF	-	-
γ-Terpinene	99-85-4	4.144±0.099	24.683±9.831	4.377±0.177	2.14[1)]	＜1	＜1

γ-Elemene	29873-99-2	18.31±0.5677	-	-	NF	-	-
γ-Curcumene	28976-68-3	163.13±10.001	-	-	NF	-	-
β-Myrcene	123-35-3	60.103±5.896	44.659±12.831	57.881±3.334	0.0166[1)]	26.903	34.868
β-Caryophyllene	87-44-5	-	2.689±0.223	17.521±2.652	0.16[1)]	＜1	1.095
β-Phellandrene	555-10-2	-	-	86.97±5.041	0.36[1)]	-	2.416
β-Pinene	127-91-3	2.248±0.097	3.609±0.464	-	36.1[1)]	＜1	-
β-Ocimene	13877-91-3	1.789±0.01	-	-	NF	-	-
β-Bisabolene	495-61-4	513.761±16.587	71.292±11.725	2.076±0.957	0.006[1)]	118.820	4.360
β-Elemene	515-13-9	30.262±3	43.405±11.831	-	0.0012[1)]	333.885	-
α-Terpinene	99-86-5	9.523±0.113	11.373±6.01	2.489±0.3443	0.08[1)]	1.422	＜1
α-Pinene	80-56-8	105.693±8.65	39.092±9.031	-	2.01[1)]	＜1	-
α-Farnesene	502-61-4	425.963±14.389	22.242±7.831	-	2[1)]	＜1	-

3-Carene	13466-78-9	-	6.442±0.947	13.682±6.565	NF	-	-
7-Ethoxycarbonylcycloheptatriene	27332-37-2	-	3.639±0.554	-	NF	-	-
α-Curcumene	644-30-4	426.502±14.432	109.517±17.052	0.861±0.1238	NF	-	-
α-Gurjunene	489-40-7	5.855±0.123	-	-	NF	-	-
α-Cubebene	17699-14-8	1.005±0.02	-	-	0.014[1)]	-	-
Perillene	539-52-6	-	2.917±0.153	-	NF	-	-
o-Cymene	527-84-4	3.686±0.089	-	14.526±0.501	0.4[1)]	-	
l-Phellandrene	99-83-2	36.68±2.002	-	-	0.2[1)]	-	＜1
1-Methyl-4-(1-methylethenyl)-Benzene	1195-32-0	2.049±0.0985	10.175±7.301	-	NF	-	-
(3E,5E)-2,6-Dimethyl-1,3,5,7-octatetracene	460-01-5	-	3.087±0.544	-	NF	-	-
(E)-3,7-Dimethylocta-1,3,6-triene	3779-61-1	4.156±0.097	33.919±5.831	20.237±6.522	NF	-	-

(E)-1,1-Dimethyl-2-(3-methylbuta-1,3-dien-1-yl)cyclopropane	68998-21-0	654.094±13.229	-	-	NF	-	-
(3E)-4,8-Dimethyl-1,3,7-nonatriene	19945-61-0	2.574±0.094	-	-	NF	-	-
(+/-)-δ-Elemene	20307-84-0	7.193±0.321	-	-	NF	-	-
Aromandendrene	489-39-4	15.948±0.788	-	-	NF	-	-
(+)-Cyclosativene	22469-52-9	15.431±0.788	-	-	NF	-	-
2-Methyltridecane	1560-96-9	1.726±0.03	-	-	NF	-	-
Cyclopropane	75-19-4	11.374±0.345	-	-	NF	-	-
Phenethyl acetate	103-45-7	-	1.194±0.123	0.512±0.105	0.2[1)]	＜1	＜1
Linalyl acetate	115-95-7	-	98.851±16.54	-	0.1[1)]	9.885	-
Isoamyl acetylacetate	2308-18-1	-	-	99.515±5.034	NF	-	-
Methyl palmitate	112-39-0	-	0.359±0.033	0.494±0.022	4000[1)]	＜1	＜1

Hexadecanoic acid ethyl ester	628-97-7	5.796±0.101	2.785±0.23	0.344±0.157	1.5[1)]	＜1	＜1
Hexanoic acid ethyl ester	123-66-0	4.156±0.089	-	20.237±0.633	0.075[1)]	＜1	2.698
Acetic acid ethyl ester	141-78-6	88.614±6.018	-	-	35.5[1)]	-	-
4-Terpinenyl ester of isobutanoic acid	78499-88-4	-	85.203±15.731	-	NF	-	-
Geranyl acetate	105-87-3	298.01±13.229	10.021±3073.6	-	0.1[1)]	1.002	-
Bornyl acetate	76-49-3	39.931±3.001	-	-	0.075[1)]	-	-
Octanoic acid ethyl ester	106-32-1	13.736±0.357	4.474±0.364	-	0.032[1)]	1.398	-
Pentanoic acid ethyl ester	539-82-2	0.353±0.008	0.439±0.053	0.63±0.337	0.025[1)]	＜1	＜1
Methyl salicylate	119-36-8	3.869±0.091	-	-	0.06[1)]	-	-
Tetradecanoic acid ethyl ester	124-06-1	1.354±0.01	-	-	NF	-	-
Ethyl 9-oxononanoate	3433-16-7	-	6.743±0.937	13.566±0.902	NF	-	-
α-Terpinyl acetate	80-26-2	-	-	13.435±0.153	1[1)]	-	＜1

Linalyl propionate	144-39-8	-	-	12.186±0.462	NF	-	-
Diisobutyl phthalate	84-69-5	-	1.367±0.12	2.406±0.544	NF	-	-
Ethyl cinnamate	103-36-6	-	1.732±0.13	4.513±0.106	0.0006[1)]	28.871	75.219
2-Hydroxy-propanoic acid ethyl ester	97-64-3	7.612±0.152	-	8.092±0.123	10[1)]	-	＜1
2-Nonanyl acetate	14936-66-4	10.153±0.203	-	-	NF	-	-
Butanedioic acid diethyl ester	123-25-1	1.037±0.03	-	-	1045[1)]	-	-
Decanoic acid ethyl ester	110-38-3	7.582±0.112	-	-	0.49[1)]	-	-
Butanoic acid ethyl ester	105-54-4	0.38±0.007	-	-	0.015[1)]	-	-
Propanoic acid ethyl ester	105-37-3	0.388±0.009	-	-	0.01[1)]	-	-
4-Methylpentyl 2-methylbutanoate	35852-40-5	1.219±0.034	-	-	NF	-	-
2-Heptyl acetate	5921-82-4	10.217±0.3643	1.608±0.1367	-	NF	-	-

Methylester of 1-Methyl-2,5-cyclohexadiencarboxylic acid	59034-54-7	1.164±0.021	-	-	NF	-	-
Citronellyl acetate	150-84-5	144.133±8.7	-	-	NF	-	-
1-Hexanol	111-27-3	-	0.83±0.073	-	0.2[1)]	＜1	-
1-Octanol	111-87-5	-	-	9.13±0.270	260[1)]	-	＜1
Eucalyptol	470-82-6	-	-	86.97±4.628	0.00026[1)]	-	3345.017
(-)-Terpinen-4-ol	20126-76-5	-	65.169±14.731	-	NF	-	-
(3Z)-3,7-Dimethyl-3,6-octadien-1-ol	5944-20-7	35.297±2.576	-	-	NF	-	-
6-Octen-1-ol, 3,7-dimethyl-, (R)-	1117-61-9	86.978±5.954	-	-	NF	-	-
(2S)-2-Heptanol	6033-23-4	8.706±0.105	-	-	NF	-	-
1-(1',1'-dimethylethyl)-2,2-dimethyl-2,3-dihydro-1H-inden-5-ol	110327-23-6	12.533±0.3643	-	-	NF	-	-

1-Propanol	71-23-8	0.68±0.004	-	-	45[1)]	-	-
Endo-Borneol	507-70-0	3.015±0.081	-	-	NF	-	-
1-Heptanol	111-70-6	-	-	3.044±0.23	0.52[1)]	-	＜1
1-Octen-3-ol	3391-86-4	-	-	42.907±11.223	NF	-	-
2-Heptanol	543-49-7	-	0.547±0.043	-	0.1[1)]	＜1	-
2-Nonanol	628-99-9	19.525±0.894	-	-	0.28[1)]	-	-
3-Methyl-1-butanol	123-51-3	30.478±2.438	-	-	0.25[1)]	-	-
α-Eudesmol	473-16-5	1.856±0.04	-	-	NF	-	-
d-Nerolidol	142-50-7	-	1.01±0.1367	-	NF	-	-
α-Terpineol	98-55-5	82.702±6.145	-	10.231±0.765	0.3[1)]	-	＜1
β-Eudesmol	473-15-4	15.398±0.7881	-	-	NF	-	-
(±)-Trans-Nerolidol	40716-66-3	18.886±0.812	-	-	1[1)]	-	-

Benzyl alcohol	100-51-6	-	1.427±0.16	7.204±0.364	5.5[1)]	＜1	＜1
Benzeneethanol	60-12-8	-	39.593±8.831	51.251±2.325	0.045[1)]	＜1	11.389
Linalool	78-70-6	61.336±5.378	424.412±31	207.67±13.558	0.0015[1)]	42.441	20.767
1-Dodecanol	112-53-8	-	-	0.623±0.12	NF	-	-
Geraniol	106-24-1	142.645±9.12	8.906±0.967	-	0.0075[1)]	11.875	-
Ethanol	64-17-5	616.554±17.23	14.498±4.321	51.401±1.696	2900[1)]	＜1	＜1
2-Methyl-1-propanol	78-83-1	1.084±0.021	0.892±0.03	-	5.25[1)]	＜1	-
2-Undecanol	1653-30-1	79.536±4.955	-	-	0.07[1)]	＜1	-
3-Octen-1-ol	18185-81-4	-	8.212±0.867	-	NF	-	-
Terpinen-4-ol	562-74-3	21.74±1.2613	-	18.702±0.1367	0.25[1)]	-	-
2,4,4-Trimethyl-3-vinylcyclopentanone	108946-79-8	0.699±0.003	-	-	NF	-	-
2-Heptanone	110-43-0	2.195±0.0985	-	-	0.068[1)]	-	-

(1S,4R)-fenchone	4695-62-9	-	8.719±0.977	-	NF	-	-
(+)-3-Thujone	471-15-8	-	8.042±0.807	1.537±0.073	NF	-	-
2,3-Octanedione	585-25-1	-	1.897±0.143	114.333±16.807	NF	-	-
2-Nonanone	821-55-6	24.716±1.2613	1.649±0.163	-	0.05[1)]	＜1	-
2-Undecanone	112-12-9	175.564±9.1617	5.169±0.667	-	0.01[1)]	5.169	-
α-Thujone	546-80-5	-	2.634±0.127	-	NF	-	-
6-Methyl-5-hepten-2-one	110-93-0	-	189.559±23.01	206.92±4.012	0.1[1)]	18.956	20.692
Hydroxyacetone	116-09-6	-	0.521±0.02	0.361±0.137	0.2[1)]	＜1	＜1
Acetoin	513-86-0	0.067±0.001	20.794±9.61	-	10[1)]	＜1	-
(E)-1-Allyl-2-(prop-1-en-1-yl)disulfane	122156-02-9	0.922±0.004	-	-	NF	-	-
2-Vinyl-4H-1,3-dithiine	80028-57-5	16.895±0.632	-	-	0.0016[1)]	-	-
3-Vinyl-1,2-dithiacyclohex-4-ene	62488-52-2	24.793±1.2613	-	-	NF	-	-

Diallyl trisulfide	2050-87-5	113.314±8.001	-	-	0.00034[1)]	-	-
Diallyl disulphide	2179-57-9	480.193±13.976	114.698±16.555	-	0.0043[1)]	266.740	-
Allyl Sulfide	592-88-1	8.924±0.153	4.178±0.44	-	0.01[1)]	4.178	-
2-Methylthiirane	1072-43-1	21.36±1.2613	64.731±15.431	-	NF	-	-
Methyl allyl disulfide	2179-58-0	5.876±0.122	1.175±0.128	-	0.00003[1)]	391.667	-
Geranial	141-27-5	-	-	246.79±8.707	0.04[1)]	-	61.698
3,7-Dimethyl-3,6-octadienal	55722-59-3	-	44.181±5.831	-	NF	-	-
Cuminaldehyde	122-03-2	-	-	3.693±0.284	0.4[1)]	-	＜1
Z-Citral	106-26-3	-	-	157.699±24.696	NF	-	-
Benzaldehyde	100-52-7	-	21.685±6.01	12.026±0.967	0.3[1)]	＜1	＜1
4-Methoxybenzaldehyde	123-11-5	-	12.249±3.321	4.86±0.334	0.1[1)]	1.225	＜1
(2E,4E)-Deca-2,4-dienal	25152-84-5	-	-	6.249±0.03	0.00003[1)]	-	2083.073

Heptanal	111-71-7	-	4.869±0.54	31.039±2.104	0.031[1)]	1.571	10.013
Decanal	112-31-2	-	2.759±0.123	7.172±0.977	0.005[1)]	5.518	14.344
Hexanal	66-25-1	-	10.277±3.021	-	0.21[1)]	＜1	-
Dimethoxymethane	109-87-5	-	12.289±3.121	-	NF	-	-
Furfural	98-01-1	0.143±0.003	1.545±0.123	1.658±0.807	0.1[1)]	＜1	＜1
Nonanal	124-19-6	-	93.977±12.731	171.295±7.465	0.0035[1)]	268.506	489.415
Hexadecanal	629-80-1	-	1.248±0.11	-	NF	-	-
Salicylaldehyde	90-02-8	-	55.576±3.01	48.192±1.687	0.00312[1)]	178.128	154.463
Citronellal	106-23-0	-	120.964±14.22	22.648±0.667	0.0035[1)]	345.610	64.710
Octanal	124-13-0	-	20.843±9.001	45.121±2.851	0.0001[1)]	2084.250	4512.108
Acetaldehyde	75-07-0	0.665±0.002	-	-	0.167[1)]	-	-
Phenol	108-95-2	-	-	0.617±0.017	5.5[1)]	-	＜1

Eugenol	97-53-0	-	21.323±5.031	19.108±0.02	0.001[1)]	213.228	191.075
Methyl Eugenol	93-15-2	-	2.424±0.111	1.366±0.392	1.25[1)]	＜1	＜1
Cis-Isoeugenol	5912-86-7	-	0.478±0.023	0.156±0.045	NF	-	-
Geranic acid	459-80-3	8.053±0.143	-	-	NF	-	-
Octanoic acid	124-07-2	3.058±0.0985	-	-	101[1)]	-	-
Benzoic acid	65-85-0	-	2.657±0.123	1.389±0.44	1220[1)]	＜1	＜1
Nonanoic acid	112-05-0	-	1.32±0.101	1.929±0.296	1.5[1)]	＜1	＜1
Acetic acid	64-19-7	31.543±1.986	10.144±6.321	2.647±0.128	120[1)]	＜1	＜1
4-Allylanisole	140-67-0	-	349.339±28.7	40.087±2.96	0.0075[1)]	465.785	53.449
Cis-Anethole	25679-28-1	-	22.836±9.831	5.089±0.478	NF	-	-
2,3,5-Trimethylpyrazine	14667-55-1	-	-	1.331±0.330	0.071[1)]	-	＜1
methylpyrazine	109-08-0	-	-	3.153±0.54	1[1)]	-	＜1

2-Amylfuran	3777-69-3	0.82±0.005	-	5.554±0.123	0.0048[1)]	-	11.571
2-Isobutylthiazole	18640-74-9	0.865±0.002	-	-	0.003[1)]	-	-
4-Ethylphenol	123-07-9	20.352±1.2613	-	-	0.01[1)]	-	-
2-Methoxy-4-(1-propenyl)phenol	97-54-1	15.821±0.754	-	-	0.01[1)]	-	-
3-Methyl-2-(2-methyl-2-butenyl)-furan	15186-51-3	-	6.626±0.77	13.42±3.021	NF	-	-
Benzothiazole	95-16-9	-	0.275±0.093	0.424±0.089	0.35[1)]	＜1	＜1

NF, Not Fond.

“-”, Not described.

[1)] Odor thresholds taken from reference(Gemert, 2003)
